# Tracking progress on environmentally sustainable healthcare in the U.S.: survey results from climate-leading hospitals

**DOI:** 10.1016/j.joclim.2025.100618

**Published:** 2025-12-23

**Authors:** Kirsty Jones, Emily Senay, Jonathan Slutzman, Michael Padget

**Affiliations:** a Ecole des hautes études en santé publique, Saint-Denis 93210, France; b Department of Environmental Health Sciences, Yale School of Public Health, New Haven, CT 06510, USA; c Department of Emergency Medicine, Massachusetts General Hospital and Harvard Medical School, Boston, MA, 02114, USA; d Center for the Environment and Health, Massachusetts General Hospital, 55 Fruit St. Boston, MA, 02114, USA

**Keywords:** Environmentally sustainable healthcare, Health organizations, Public reporting

## Abstract

**Introduction:**

In the absence of uniform regulations, the outsized environmental impacts of the U.S. healthcare sector conflict with its mandate to improve and protect population health. This study sought to assess approaches to environmental sustainability and transparency among early-adopters of sustainable healthcare initiatives, to understand the example they might set for the broader industry.

**Methods:**

We identified signatories of the Department of Health and Human Services’ Healthcare Sector Climate Pledge as a likely group of early adopters, to whom we distributed a survey on goals, progress, reporting, and organizational approaches pertaining to various facets of environmental sustainability, in addition to reviewing their public websites.

**Results:**

Among the sample population, both progress and transparency were lacking. Survey response rates were poor and demonstrated no consistent pattern in approach to sustainability. Survey respondents showed greater progress on decarbonization as compared with renewable energy, water usage, and waste reduction.

**Conclusions:**

Though limited by a poor response rate, the survey findings demonstrated little reason to expect efficient progress in the U.S. health sector across four main areas (carbon emissions, renewable energy usage, water usage, and waste management). Findings demonstrate limited secondary evidence of environmental improvement, including publicly stated goals, transparent sustainability data, and contact information for sustainability staff. The results did not provide sufficient successful examples to create a blueprint for wider efforts. More progress is required to meet the environmental aspirations of the health sector, and protect public and planetary health.

## 1 Introduction

Climate change is a significant threat to humanity, through environmental, financial, and health harms [1]. Although a wide array of industries have begun to incorporate climate risk reduction as a priority, the healthcare industry lags [[2], [3], [4]].

The environmental impacts from the U.S. health system are substantial; per capita carbon emissions are the highest of any health system globally [[5], [6], [7]]. These environmental costs, coupled with high U.S. health spending (financial costs), are not associated with improvements in health outcomes or access at either the state or the national level [8,9]. Rather, elevated emissions contribute to worse health outcomes, creating a vicious cycle [8,10].

Pursuing more sustainable healthcare practice is imperative to upholding the ethical principles under which the health industry operates. New policies that protect and promote health by reducing the negative environmental impacts of health care provision without reducing the quality or accessibility of care are needed [[11], [12], [13]].

Organizations such as the National Academy of Medicine and the Joint Commission have begun advancing work in this area, providing guidance for early adopters [14,15]. Other fee-based membership organizations seek to incentivize action through pledges, sustainability training programs, and recognition awards [16,17].

Progress on sustainability requires clear goals, transparent, verified and actionable data, and effective solutions. Despite the growing interest, there is no industry-wide consensus on how to achieve environmental sustainability in healthcare. No standard reporting or accountability mechanism exists [18], and a recent survey of clinicians, clinical leaders, and executives at organizations around the world [19] demonstrated poor effort and progress on sustainability.

To gain insight into environmental initiatives in the health sector and support the development of a clear roadmap and environmental benchmarks, we conducted a survey of U.S. health organizations with a public commitment to sustainability through the U.S. Department of Health and Human Services’ (HHS) Healthcare Sector Climate Pledge. The pledge was a voluntary commitment by healthcare organizations to improve climate resilience and carbon emissions. We sought to assess the approaches of these organizations toward improvements in key sustainability areas including but not limited to carbon emissions reduction. Secondary aims included assessing the extent to which these approaches are transparently represented to the community and identifying the resources attributed for this work within each organization.

## 2 Methods

### 2.1 HHS climate pledge

In 2022, HHS launched the Health Care Sector Climate Pledge. While no formal accountability or reporting mechanisms were included in this voluntary pledge, signing organizations agreed to a number of greenhouse gas emission reduction goals, the designation of an executive-level lead for environmental work, and the completion of a scope 3 greenhouse gas inventory by the end of 2024, among other commitments [20].

For this study we consider pledge signatories listed on the HHS website in March 2024 as having made a strong public commitment to sustainability and as potential leaders in this work.

Following completion of data collection for this study, information about the HHS Climate Pledge was removed from federal websites in February 2025 after the change in administration and is no longer publicly accessible [21].

### 2.2 Data collection

We gathered data from eligible organizations using website audits and surveys between the 8th of March 2024 and the 18th of February 2025.

### 2.3 Website audit

First, we reviewed the public-facing websites of pledge signatories to understand how their environmental sustainability efforts were represented publicly. The audit was repeated twice for completeness, and finalized in October 2024. Relevant information was sought through direct navigation of the websites, internet search (using Google), and intra-site search.

We collected the following datapoints as binary variables from the websites: any mention of sustainability; a dedicated webpage; a contact directly related to sustainability; goals relating to sustainability; additional sustainability goals exceeding those required by the pledge; any public reporting of sustainability data. These were required to be featured on a permanent, public-facing segment of the website; isolated anecdotal media articles were not included.

### 2.4 Survey

Along with our website review, we developed a 92-item questionnaire using REDCap software [22,23] to assess the participants’ approaches to sustainability. Existing questionnaires from organizations such as the American Hospital Association and the HHS itself were consulted during the development phase, with the aim of facilitating future comparison with these larger datasets should they become available.

Carbon emissions were characterised according to the standard three scopes as defined by the Greenhouse Gas Protocol [24]. These include scope 1 (direct emissions from operations, e.g., onsite fossil fuel combustion, operated vehicles), scope 2 (emissions from purchased energy, e.g. electricity and steam), and scope 3 (other indirect emissions such as from the supply chain, construction and renovation, staff travel) [24,25]. All emissions are accounted for under this framework, but for a more granular understanding of where efforts are being made, we also explored the following related areas of interest:

• Renewable energy usage
• Water conservation
• Waste management
• Supply chain management
• Organizational structure

**Fig. 1:**
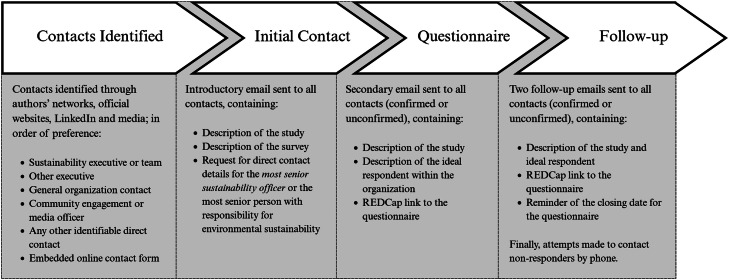
Points of Contact with Target Organizations Following the identification of contacts, we made multiple attempts at confirming contacts and providing access to the survey. Attempts were also made to follow up via email and phone to encourage completion of the survey.

The HHS signatories were identified and contacted as described in Fig. 1. Contacts received a link to the REDCap landing page, containing a detailed description of the study and an electronic mechanism for giving explicit consent, which was required to access the questionnaire.

A collapsible structure to the questions ensured that respondents were faced with in-depth questions only where relevant to their organizations’ efforts. Submissions could also be revisited repeatedly throughout the data collection period.

## 3 Results

### 3.1 Review of websites

Table 1 demonstrates the results of the website review; whilst over half of the pledge signatories made some mention of sustainability on their website, goals (either those within or beyond the scope of the pledge), progress, and relevant contacts within the team were much less commonly disclosed.

**Table 1:** Audit of sustainability-related features publicly visible on HHS climate pledge signatories’ websites, with comparison between survey-responders (*n* = 14) and survey-non responders (*n* = 71).

Feature	Total ( %)		Responders ( %)	Non-responders ( %)	
Mention of sustainability	53 (62)		9 (64)	44 (62)	
Sustainability webpage	50 (59)		9 (64)	41 (58)	
Sustainability contact	17 (20)		2 (14)	15 (21)	
Publicly stated goals	44 (52)		9 (64)	35 (49)	
Goals beyond the pledge mentioned	35 (41)		6 (43)	29 (41)	
Public reporting of progress	38 (45)		7 (50)	31 (44)	

### 3.2 Survey response

Of the 86 organizations listed on the HHS website at the time of initiating the study (April 2024), contacts were identified for 78. In total, 14 organizations responded, 19 % of those contacted.

#### 3.2.1 Characteristics

Table 2 presents the characteristics of the survey respondents. Only four organizations employed a full-time chief sustainability officer; these organizations were evenly distributed across the range of organization sizes, with no correlation between bed number and employment of a full-time sustainability officer.

**Table 2:** Organization characteristics and sustainability resources of survey respondents.

Characteristic	n ( % of respondents)
Not-for-profit status	13 (93)
Presence of sustainability team	10 (71)
Participation in fee-based sustainability awards	7 (50)
	**mean (std)**
Number of beds	3983 (4458)
FTE working on sustainability	4.1 (3.5)
Funded FTE working on sustainability	3.0 (3.7)

In four cases, the main responsibility for sustainability duties were held by other executives such as the CFO. The sustainability officers most commonly had backgrounds in environmental science, administration and logistics, nursing, or finance. Although 9 (64 %) of the responding organizations reported employing structural motivators such as top-down mandates, personal motivations were most frequently reported (79 %) as a driving force for staff.

#### 3.2.2 Activity

**Fig. 2:**
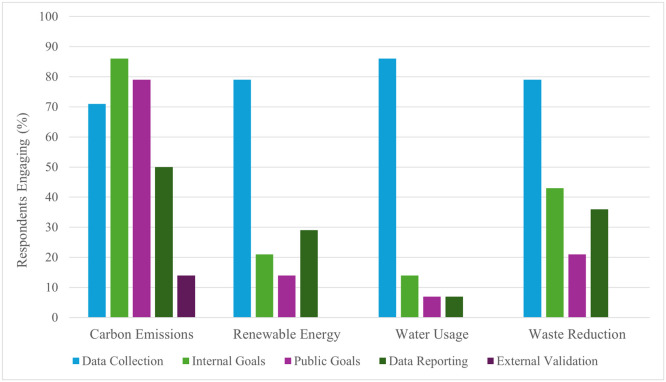
Approach to Sustainability Percentage of responding health organizations engaging in data collection, creating internal goals, declaring public goals, reporting data, and seeking external validation of data across four areas of sustainability (carbon emissions, renewable energy, water usage, and waste reduction).

As demonstrated in Fig. 2, rates of data collection exceeded 70 % across areas.

Twelve respondents (86 %) had established either public or internal goals relating to scope 1 and 2 carbon emissions – in 8 cases (57 %), goals included scope 3. For both scope 1 and 2, 9 (75 %) declared being on track to meet current goals; for scope 3, only 3 of 8 with goals (37 %) were on track.

For carbon emissions, 12 (86 %) had set goals; 7 (50 %) reported data. Per Fig. 2, less than half of respondents had set goals or reported data on renewable energy, water usage, or waste reduction. External validation of data was extremely infrequent.

In relation to renewable energy usage, 4 of the 5 (80 %) respondents with established goals were on track to meet them. Only 1 of 2 (50 %) respondents with water-usage goals, and 4 of 7 (57 %) respondents with waste-management goals were on track to meet them.

#### 3.2.3 Supply chain

Though 4 (29 %) respondents reported valuing environmental sustainability in their purchasing decision-making process, only two applied any contractual standards or environmentally-preferred designations to suppliers.

## 4 Discussion

Despite a poor survey response rate, we were able to gather valuable data into the activities of hospitals with a stated commitment to environmental sustainability. These data were gathered via website audits and surveys, and related to several key areas of interest identified in existing literature, including carbon emissions, water usage, pollution, and waste production [[26], [27], [28], [29]].

Overall, our results showed lack of progress in several keys areas of sustainability including measurement, goal setting, transparent third party verified disclosures, and resource allocation. Audit of websites of all eligible institutions showed lower proportions of engagement across criteria than was expected for the signatories of the HHS pledge. Institutions responding to our questionnaire reflected higher levels of engagement across areas than non-respondents, though they were not more likely to have a publicly listed sustainability contact. Both groups showed significant gaps, and there was insufficient progress to support a roadmap or benchmarking, as was our initial objective. Despite the recent interest in the topic, our results mirror those of similar studies done in the last ten years [4].

Some variation was noted between environmental topics, with more progress in the areas of greenhouse gases. Survey responses showed notably higher proportions of respondents on track to meet greenhouse scope 1 and 2 goals than scope 3. Scope 1 and scope 2 emissions may be simpler to manage at the organization-level [30,31]. Scope 3 emissions are much more complex, requiring detailed understanding of embedded carbon in purchased products, supply chain, and patient behavior. Managing these emissions is often more difficult and can include trying to influence suppliers [30,31]. In our survey, only 27 % of respondents reported efforts in this area.

Slower progress in scope 3 is unsurprising, but is likely to have important environmental consequences, with scope 3 emissions representing around 70 % of health sector emissions [25]. In the areas of renewable energy, water, and waste management, data collection was widespread. This did not always translate to action, however as goal setting was rare for these topics.

Our results suggest low prioritization of sustainability among healthcare organizations, consistent with the NEJM survey’s [19] finding of conflicting organizational priorities as the most common barrier to sustainability. This theme is illustrated by a general under-resourcing of sustainability. Within our sample of respondents, only 71 % had an established team working on environmental sustainability. Additionally, given the relatively large organizations involved, teams appeared to be small, with much of the work being undertaken on a voluntary basis alongside other responsibilities. Only four of the responding organizations had even one full time sustainability officer. Overall, the survey findings portray small, underfunded teams working on a very broad and complex issue.

We also observed a lack of consistency across the health system. This was demonstrated well among the relatively small group of sustainability officers, where no clear pattern emerged regarding their training or the apparent scope of their role. Some organizations relied upon a single chief sustainability officer. Some filled other roles under which sustainability was one of several priorities – for example, supply chain or investment. Their backgrounds and training were also diverse, without any clear unifying feature.

Although sustainability is a complex issue, requiring expertise from a variety of perspectives, this snapshot speaks to a lack of standardization, and to a lack of clear guidance. No clear image emerges of who's doing the work, how those people are arranged within the organization, or how they're supported to give sufficient priority to sustainability, where conflicting priorities exist. This is not an isolated finding; progress within the health sector is hampered by both a lack of clear definition for environmental sustainability, and a lack of accepted standards of measurement [18].

Across the various elements of the survey, clear patterns failed to emerge around how these complex issues were being approached. Without clear guidance, a roadmap is unlikely to develop organically or efficiently in an industry facing unique operational and ethical challenges.

The fragmented structure of the U.S. healthcare system may play a part in this heterogeneity. Unified health systems such as the National Health Service in the UK are managed more centrally at a national level and can facilitate change through nationally-implemented initiatives. As a result of a clear political commitment and the system structure, the NHS is considered an international leader in health system sustainability [32].

The survey findings also demonstrated a lack of transparency. Carbon emissions goals were frequently reported publicly, possibly attributable to the influence of the HHS pledge itself. However, less than half of respondents published any data relating to carbon emissions. The other areas of interest fared worse. Less than 40 % publicly reported any goals or data relating to renewable energy, water usage, or waste reduction. External validation of data was essentially non-existent.

Overall, only half of pledge signatories had published any goals *or* data on their work in environmental sustainability. The lack of transparency is a cause for concern as transparency is the foundation of the collective efforts toward all sustainability initiatives. Furthermore, as rollbacks in federal climate policies weaken oversight, robust anti-greenwashing safeguards become more essential than ever to prevent institutions from making false sustainability claims without accountability or regulatory scrutiny [33]. Recent works have described the importance of transparency in sustainable healthcare, and demonstrated its under-appreciation in the current literature, and in the industry [4,34].

Sustainability accounting has become a fixture of corporate governance both in the U.S. and internationally, ensuring environmental impacts are tracked, improved, and critically reported. In addition to increasing accountability and trust for all stakeholders, sustainability accounting reduces the risk of inadvertent or intentional greenwashing [4,34,35]. Robust collection and validation of data, planning and implementation of interventions, and tracking and reporting of outcomes are all key to the rapid progress necessary to mitigate an impending climate catastrophe [4,34,35].

In the case of our survey, the HHS pledge did not specify how or where organizations were to disclose progress or accountability or mechanisms to ensure accuracy of self-reported data through third party verification as is standard in sustainability practice outside the healthcare sector. It must be acknowledged that pledges are ineffective without mandated, third-party verified data because voluntary commitments alone lack the accountability needed to drive real change. Lack of verified public data often fosters greenwashing, where sustainability narratives are used for reputational gain without meaningful action [36].

While our results indicate slow progress on sustainability among our study population and survey respondents, we believe progress in the wider population of hospitals is likely to be much lower. Survey respondents outperformed the total population included in our study across almost every metric, using both survey and audit data. Respondents also rated higher across a number of domains captured in an international survey of hospitals performed among NEJM Catalyst Insights council members [19]. These higher scores reflect greater engagement among survey respondents and likely relatively slower progress among organizations beyond the HHS pledge.

Future progress on this issue is very likely to be impacted by the current political environment. Our study used signers of the HHS pledge as a sampling frame. The public commitment of this pledge likely played a role in advancing work done at these institutions and the closing of the HHS Climate Office and removal of the Climate Pledge have reversed much of the political momentum while removing resources available to institutions interested in pursuing this work. The full impact of these decisions on ongoing or planned work at health institutions as well as at other notable groups working in this space such as the Joint Commission and CMS is yet to be seen. As a result, more than ever, the sector is in need of guidance. Early adopters of sustainable healthcare such as the pledge signatories could play a crucial role in guiding the rest of the industry; however our findings demonstrate that they may not yet be ready to do so.

Some positive initiatives emerged, such as the use of structural factors to encourage progress in challenging but impactful areas. For example, executive support for dedicated teams may reduce conflicting organizational priorities, a major obstacle [19]. Similarly, contractual standards for suppliers may be valuable in reducing complex scope 3 emissions. However, at present, supplemental expertise may be required from international examples like the NHS.

### 4.1 Limitations

Several limitations impacted the generalizability of our study results. Firstly, survey results are limited due to the poor survey response rate of just under 20 %. Although a series of attempts at contact were made, as described above, in approximately 80 % of cases, an immediately relevant contact was never identifiable. This difficulty does, however, also serve to further demonstrate a main finding of this study – a lack of accessibility, and further, a lack of transparency. Although attempts were made as described above to streamline the process for respondents, questionnaire-specific factors such as the length may also have deterred potential respondents.

Secondly, respondents were not random and may well represent a biased sample of HHS Pledge members and the U.S. health system in general. Additionally, our survey identified initiatives and activities associated with environmental progress but did not measure direct markers such as carbon emissions or reductions thereof.

### 4.2 Moving forward

Future research should aim to collect validated data from these organizations, where it is available, to independently assess progress, the importance of which has been noted by other authors [4,35]. Additionally, it would be valuable to perform an in-depth assessment of the barriers to progress, and to disclosure of information, to understand where improvements might be made. For instance, it should be explored if rather than relying on unenforceable pledges—which are often unrealistic and prone to aspirational overreach—healthcare systems might better focus on setting realistic, science-aligned goals specific to their institution and publicly sharing third-party verified data to demonstrate measurable progress. In this context, verified, transparent data would serve as the minimum requirement for any serious climate leadership initiative or declaration. Promoting a culture of evidence-based progress rather than symbolic commitment also would better align with the healthcare quality movement where quantified outcome metrics are the norm [26].

## 5 Conclusion

The current study aimed to assess progress and transparency among healthcare systems leading in sustainability, with the expectation that they might represent a group of early-adopters of sustainable healthcare, from whom the broader U.S. healthcare industry can take guidance, to facilitate rapid progress in the absence of unified regulations. Limited by a poor response rate, the survey findings demonstrated little progress across four main areas (carbon emissions, renewable energy usage, water usage, and waste management). Additionally, transparency was very limited, with very little reporting, and almost no external validation of data. If this group is representative of – or as expected, ahead of – the U.S. average, with little consistency in approach, little progress, and very minimal transparency, the industry is not well supported to make the rapid progress required to fulfil its ethical and organizational imperative to protect the health of the population.

## Funding

This research did not receive any specific grant from funding agencies in the public, commercial, or not-for-profit sectors.

## CRediT authorship contribution statement

**Kirsty Jones:** Writing – review & editing, Writing – original draft, Project administration, Methodology, Investigation, Formal analysis, Data curation, Conceptualization. **Emily Senay:** Writing – review & editing, Conceptualization. **Jonathan Slutzman:** Writing – review & editing, Supervision, Conceptualization. **Michael Padget:** Writing – review & editing, Supervision, Project administration, Methodology, Investigation, Formal analysis, Data curation, Conceptualization.

## Declaration of competing interest

The authors declare that they have no known competing financial interests or personal relationships that could have appeared to influence the work reported in this paper.

## Appendix Supplementary materials

Image, application 1
