# Supplementary material for: Tracking progress on environmentally sustainable healthcare in the U.S.: survey results from climate-leading hospitals
Source: J Clim Chang Health. 2025 Dec 23;27:100618. doi: 10.1016/j.joclim.2025.100618 (PMC13184506; doi:10.1016/j.joclim.2025.100618)
Supplement: Supplementary file 1 [file mmc1.pdf]

# Environmental Sustainability among HHS Pledge Signatories

As a signatory of the White House-HHS Health Sector Climate Pledge, your organization is aware of the complex interplay between the healthcare sector and the climate. Sustainable Healthcare is a new and emerging field; no one-size-fits-all approach exists, to guide the sector toward a more sustainable model of top-quality care. As an early leader in this space, the work done by your organization is both a marker of progress, and a valuable example to the wider healthcare sector.

This survey aims to examine broadly the progress made by the group of HHS signatory organizations, as well as the approaches to the broad challenge of sustainable healthcare.

The results of the survey will be collated for the purpose of scientific publication on the approaches and progress among the healthcare sector. The results will be instrumental in identifying best practices and identifying gaps in the field. No organizations, hospitals, or individuals will be named.

This survey is expected to take, on average, 20 to 30 minutes, depending on your activities. By completing and submitting the survey, you consent on behalf of your organization to the use of anonymised and aggregated data for the purposes of scientific publication.

---

Name ______________________

---

Email Address ______________________

---

Phone Number ______________________

---

Which healthcare system do you represent? ______________________

---

Approximately how many staffed beds does the organisation represent? ______________________

---

What is the ownership structure of the organisation?

- ◯ Government- federal
- ◯ Government- nonfederal
- ◯ Nongovernment- not-for-profit
- ◯ Investor-owned- for-profit
- ◯ Other

---

What is your role within the organisation? ______________________

## External Declarations

| | |
|---|---|
| Has your organisation made other publicly-declared commitments in addition to the HHS Pledge? E.g. UN Race to Zero, Laudato Si' Action Platform | ◯ Yes<br>◯ No |
| Please list the additional commitments made in addition to the HHS Pledge?<br>E.g. UN Race to Zero, Laudato Si' Action Platform | __________________________________ |
| What are the sources of these commitments? | ☐ Voluntary<br>☐ Federal government<br>☐ State government<br>☐ Local government<br>☐ N/A |
| Has your organization signed the UN Global Compact? https://unglobalcompact.org/ | ◯ Yes<br>◯ No |
| Does your organisation's annual corporate report include sustainability data? | ◯ Yes<br>◯ No |
| Is it verified by any third party? | ◯ Yes<br>◯ No |

## Carbon Emissions

| | |
|---|---|
| Does your organisation have existing publicly-declared or internal goals relating to carbon (greenhouse gas) emission reductions? (choose all answers that apply) | ☐ Yes: they have publicly declared goals relating to carbon emissions.<br>☐ Yes: they have internal goals relating to carbon emissions.<br>☐ They are in the process of establishing publicly-declared goals.<br>☐ They are in the process of establishing internal goals.<br>☐ No: they have not established goals in this area. |
| To which Scopes do the established goals apply? | ☐ Scope 1<br>☐ Scope 2<br>☐ Scope 3 |
| To which Scopes do the planned goals apply? | ☐ Scope 1<br>☐ Scope 2<br>☐ Scope 3 |
| What are your organization's publicly-reported goals related to carbon emissions? | ______________________________ |
| Where is your organization publicly declaring those goals? (ESG reports, sustainability frameworks, marketing materials, press releases, awards, website, other) | ______________________________ |
| What are your organization's internal goals related to carbon emissions? | ______________________________ |
| Please describe the current progress of the organization on carbon emissions<br>Scope 1: | ◯ Exceeding or on track to exceed most ambitious carbon emission goals (can be either internal or external)<br>◯ Meeting or on track to meet goals<br>◯ Not yet meeting goals<br>◯ Don't know |
| Please describe the current progress of the organization on carbon emissions<br>Scope 2: | ◯ Exceeding or on track to exceed most ambitious carbon emission goals (can be either internal or external)<br>◯ Meeting or on track to meet goals<br>◯ Not yet meeting goals<br>◯ Don't know |
| Please describe the current progress of the organization on carbon emissions<br>Scope 3: | ◯ Exceeding or on track to exceed most ambitious carbon emission goals (can be either internal or external)<br>◯ Meeting or on track to meet goals<br>◯ Not yet meeting goals<br>◯ Don't know |
| Does your organization track carbon emissions? | ◯ Yes<br>◯ No |
| Which Scopes does it track? | ☐ Scope 1<br>☐ Scope 2<br>☐ Scope 3 |

| | |
|---|---|
| Does your organization use a standard protocol or specific tools to track carbon emissions? Please describe: | __________________________ |
| If your organization does not use standard tools and protocols, how does it track carbon emissions? | __________________________ |
| Does your organization publicly report data related to carbon emissions? | ◯ Yes<br>◯ No |
| What carbon emissions data are publicly reported? | ________________________________ |
| Where are those data reported? | ________________________________ |
| Are these data externally validated by a third-party? | ◯ Yes<br>◯ No |

## Renewable Energy Usage

| | |
|---|---|
| Does your organisation have existing publicly-declared or internal goals relating to renewable energy usage? | ☐ Yes: they have publicly declared goals relating to renewable energy.<br>☐ Yes: they have internal goals relating to renewable energy.<br>☐ They are in the process of establishing publicly-declared goals.<br>☐ They are in the process of establishing internal goals.<br>☐ No: they have not established goals in this area. |
| What are the publicly-reported goals? | __________________________________ |
| Where is your organization publicly declaring those goals? | __________________________________ |
| What are the internal goals? | __________________________________ |
| Please describe the current progress of the organization on renewable energy usage: | ◯ Exceeding or on track to exceed most ambitious goals (can be either internal or external)<br>◯ Meeting or on track to meet goals<br>◯ Not yet meeting goals<br>◯ Don't know |
| Do you collect data on renewable energy usage? | ◯ Yes<br>◯ No |
| Do you publicly report data on renewable energy usage? | ◯ Yes<br>◯ No |
| What data are publicly reported? | __________________________________ |
| Where are those data publicly reported? | __________________________________ |
| Are these data externally validated by a third-party? | ◯ Yes<br>◯ No |

## Water Usage

| | |
|---|---|
| Does your organisation have existing publicly-declared or internal goals relating to water usage? | ☐ Yes: they have publicly declared goals relating to water usage.<br>☐ Yes: they have internal goals relating to water usage.<br>☐ They are in the process of establishing publicly-declared goals.<br>☐ They are in the process of establishing internal goals.<br>☐ No: they have not established goals in this area. |
| What are the publicly-reported goals? | __________________________________ |
| Where is your organization publicly declaring those goals? | __________________________________ |
| What are the internal goals? | __________________________________ |
| Please describe the current progress of the organization on water usage: | ◯ Exceeding or on track to exceed most ambitious goals (can be either internal or external)<br>◯ Meeting or on track to meet goals<br>◯ Not yet meeting goals<br>◯ Don't know |
| Do you collect data on water usage? | ◯ Yes<br>◯ No |
| Do you publicly report data on water usage? | ◯ Yes<br>◯ No |
| What data are publicly reported? | __________________________________ |
| Where are those data publicly reported? | __________________________________ |
| Are these data externally validated by a third-party? | ◯ Yes<br>◯ No |

## Waste Management

Does your organisation have existing publicly-declared or internal goals relating to waste management?

- ☐ Yes: they have publicly declared goals relating to waste management.
- ☐ Yes: they have internal goals relating to waste management.
- ☐ They are in the process of establishing publicly-declared goals.
- ☐ They are in the process of establishing internal goals.
- ☐ No: they have not established goals in this area.

What are the publicly-reported goals?

__________________________________

Where is your organization publicly declaring those goals?

__________________________________

What are the internal goals?

__________________________________

Please describe the current progress of the organization on waste management:

- ◯ Exceeding or on track to exceed most ambitious goals (can be either internal or external)
- ◯ Meeting or on track to meet goals
- ◯ Not yet meeting goals
- ◯ Don't know

Do you collect data on waste management?

- ◯ Yes
- ◯ No

Do you publicly report data on waste management?

- ◯ Yes
- ◯ No

What data are publicly reported?

__________________________________

Where are those data publicly reported?

__________________________________

Are these data externally validated by a third-party?

- ◯ Yes
- ◯ No

## Purchasing and Supply Chain

Do you have contractual standards or benchmarks your organisation requires from suppliers or service providers for the following?

- ☐ Carbon emissions
- ☐ Utilisation of renewable energy
- ☐ Impact on air and water pollution
- ☐ Impact on deforestation
- ☐ Impact on biodiversity
- ☐ Impact on stressing water supplies
- ☐ Waste and management of hazardous materials
- ☐ Waste impact of their final product and reprocessing potential

Do you have an "environmentally-preferred" designation for suppliers or service providers?

- ◯ Yes
- ◯ No

Please describe the targets or goals that the supplier must meet to achieve this designation.

__________________________________

How is this designation used?

__________________________________

When was this designation established?

__________________________

Is this designation publicly available or visible to the public?

- ◯ Yes
- ◯ No

Is this designation visible to other suppliers?

- ◯ Yes
- ◯ No

Is the environmental impact of individual products incorporated into purchasing decisions?

- ◯ Yes
- ◯ No

How is this incorporated into purchasing decisions?

__________________________________

## Organization

| | |
|---|---|
| Who is the highest level officer in the organization with environmental sustainability responsibility? | __________________________________ |
| What is that person's background, training and expertise? | __________________________________________ |
| Where are they located within the organization? | __________________________________ |
| What proportion of their time is attributed to environmental sustainability? | __________________________________ |
| Please describe the organizational structure with regard to environmental sustainability. | __________________________________________ |
| Is there a dedicated department or team working on environmental sustainability? | ◯ Yes<br>◯ No |
| How many FTE (paid or unpaid) are working on environmental sustainability within the organization? | __________________________________ |
| Are there plans to change these FTE? | ◯ Yes (increase)<br>◯ Yes (decrease)<br>◯ No |
| How many FTE are funded to work on environmental sustainability? | __________________________________ |
| What are the main factors motivating progress for staff working on environmental sustainability? | ☐ Explicit organisational goals and accountability<br>☐ Top-down mandates<br>☐ Personal interest<br>☐ Other |
| What are the sources of expertise used to develop environmental sustainability strategy within the organization? | ☐ Internal<br>☐ External |
| Do you participate in sustainability awards programs? | ◯ Yes<br>◯ No |
| Which ones? | __________________________________________ |
| Are the award programs fee-based? | ☐ Yes<br>☐ No<br>☐ Some are fee-based, some are not |
| Are data associated with awards verified by third-parties? | ◯ Yes<br>◯ No |
| Are the data associated with the awards publicly shared? | ◯ Yes<br>◯ No |

| | |
|---|---|
| Are awards used in marketing materials? | ◯ Yes<br>◯ No |
| Do you have specific goals in other areas not mentioned, which are important to your sustainability efforts? | __________________________________ |
| Do you report data in other areas not mentioned, which are important to your sustainability efforts? | __________________________________ |

**Thank you for the time taken to complete this questionnaire. As this is a complex topic, the depth of which can not entirely be captured in a brief questionnaire, we plan to conduct follow-up interviews with a small subset of respondents. These interviews, of less than an hour's duration, would further explore the specific foci, goals, mechanisms, and progress of organisations on environmental sustainability.**
**Please indicate below whether you are willing to be contacted for an interview via video call, at a time convenient to you. From those willing, a random sample will be selected.**

| | |
|---|---|
| Would you be open to conducting a brief follow-up interview with the researchers via video call, for further clarification and elaboration? | ◯ Yes<br>◯ No |
| Please provide the best contact information. | ________________________________________ |
